# Inhibition of PI4K IIIα radiosensitizes in human tumor xenograft and immune-competent syngeneic murine tumor model

**DOI:** 10.18632/oncotarget.22778

**Published:** 2017-11-30

**Authors:** Younghee Park, Ji Min Park, Dan Hyo Kim, Jeanny Kwon, In Ah Kim

**Affiliations:** ^1^ Department of Radiation Oncology, Graduate School of Medicine, Seoul National University, Seoul, Republic of Korea; ^2^ Medical Science Research Institute, Seoul National University Bundang Hospital, Seongnam, Republic of Korea

**Keywords:** phosphatidylinositol 4-kinase IIIα, radiosensitivity, immune checkpoint blockade

## Abstract

Phosphatidylinositol (PI) 4-kinase (PI4K) has emerged as a potential target for anti-cancer treatment. We recently reported that simeprevir, an anti-hepatitis C viral (HCV) agent, radiosensitized diverse human cancer cells by inhibiting PI4K IIIα *in vitro*. In this study, we investigated the radiosensitizing effect of simeprevir in an *in vivo* tumor xenograft model and the mechanism of its interaction. The immune modulatory effect of PI4K IIIα was evaluated in an immune-competent syngeneic murine tumor model.

In *in vivo* xenograft models using BT474 breast cancer and U251 brain tumor cells, inhibition of PI4K IIIα induced by simeprevir combined with radiation significantly delayed tumor growth compared to either treatment alone. PI4K IIIα inhibition led to eversion of the epithelial-mesenchymal transition as suggested by decreased invasion/migration and vascular tube formation. Simeprevir down-regulated PI3Kδ expression and PI3Kδ inhibition using RNA interference radiosensitized breast cancer cells. PI4K IIIα inhibition enhanced the radiosensitizing effect of anti-programmed death-ligand 1 (PD-L1) and decreased the expression of PI3Kδ, phosphorylated-Akt, and PD-L1 in breast cancer cells co-cultured with human T-lymphocytes. The immune modulatory effect *in vivo* was evaluated in immune-competent syngeneic 4T1 murine tumor models. Simeprevir showed significant radiosensitizing effect and immune modulatory function by affecting the CD4(+)/CD8(+) ratio of tumor infiltrating lymphocytes.

These findings suggest that targeting PI4K IIIα with an anti-HCV agent is a viable drug repositioning approach for enhancing the therapeutic efficacy of radiation therapy. The immune regulatory function of PI4K IIIα via modulation of PI3Kδ suggests a strategy for enhancing the radiosensitizing effect of immune checkpoint blockades.

## INTRODUCTION

Phosphatidylinositol 4-phosphate (PI4P), produced by PI 4-kinase (PI4K), is a common substrate for both the phospholipase C (PLC)/protein kinase C (PKC) pathways and PI 3-kinase (PI3K)/Akt pathways responsible for diverse cell functions and pathogenesis [1]. Four isotypes of PI4K, PI4K type II (α- and β-forms) and type III (α- and β-forms) have been identified and the involvement of PI4K IIIα in various cancers have been reported [2]. PI4K IIIα is associated with more invasive phenotypes in pancreatic cancer [3] and is associated with poor prognosis in hepatocellular carcinoma [4]. A recent study reported that upregulation of PI4K IIIα was associated with the migration and invasion of prostate cancer cells [5].

Recently, we reported that simeprevir, an anti-HCV agent, revealed the inhibitory effects of PI4K IIIα in various cancer cell lines and a significant radiosensitizing effect [6]. Inhibition of PI4K IIIα by simeprevir resulted in down-regulation of p-PKC and p-AKT, suggesting that simeprevir is an effective anti-cancer agent that simultaneously inhibits two important pathways known to be involved in both tumorigenesis and treatment resistance. Moreover, simeprevir prolonged γH2AX foci and down-regulated phospho-DNA-PKcs after irradiation, suggesting that the radiosensitizing effects were mediated through impaired nonhomologous end-joining repair. These results suggested the successful drug repositioning of simeprevir as a new anti-cancer agent. Based on these results, we investigated the radiosensitizing effect of PI4K IIIα inhibition in *in vivo* tumor models and evaluated the mechanisms of radiosensitization in this study.

Cancer immunotherapy has been widely investigated in various cancers [7]. Among the several target molecules, programmed death-1 (PD-1) and programmed death-ligand 1 (PD-L1) are important molecules currently under investigation and the radiosensitizing effects of these immune checkpoint blockades have been observed [8–10]. Although the mechanisms of immune checkpoint blockades are not fully understood, the PI3K/Akt pathway has been suggested to be closely involved in the immune modulatory effect of the PD-1/PD-L1 pathway [11–13].

Among the different isotypes of PI3K, PI3Kδ is mainly found in leukocytes and plays essential roles in the development and function of immune cells [14, 15]. The immune modulatory function of PI3Kδ has been widely investigated and PI3Kδ may be a therapeutic target for hematologic malignancies [16–19]. Recent studies reported the high expression of PI3Kδ in various solid tumors and anti-tumor effect of PI3Kδ inhibition [20, 21]. In this study, we investigated the effect of PI4K IIIα inhibition on the expression of PI3Kδ and PD-L1 to evaluate the possible role of PI3Kδ inhibition as a strategy to enhance the effects of immunotherapy.

## RESULTS

### *In vivo* tumor growth delay with PI4K IIIα inhibition and irradiation

**Figure 1:**
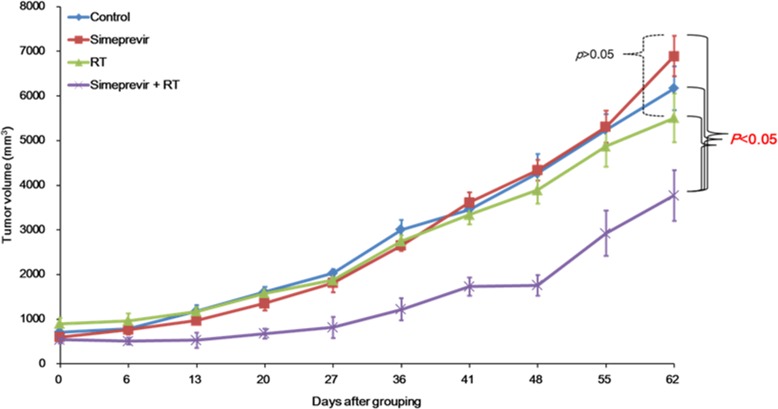
Radiosensitizing effect of simeprevir in *in vivo* human breast tumor xenograft models Tumor volume curves of each group; control, simeprevir alone, radiation alone, and simeprevir+radiation. Tumor growth was significantly delayed in the group treated with simeprevir plus radiation in BT474 breast cancer model (P < 0.05). No significant differences between control, simeprevir alone, and radiation alone were found.

**Figure 2:**
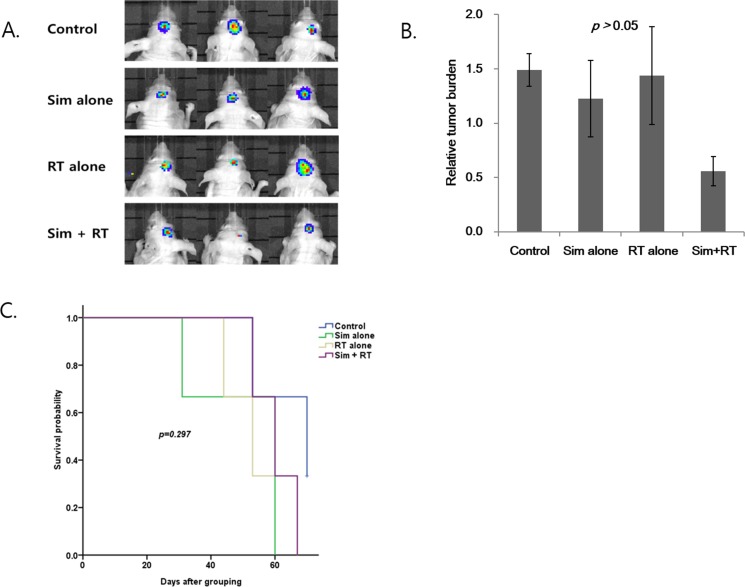
Radiosensitizing effect of simeprevir in *in vivo* human brain tumor xenograft model **(A)** Representative bioluminescence images of human brain tumor xenograft in nude mouse when all 3 mice were alive. **(B)** The relative tumor burden was lower in the group treated with simeprevir and radiation compared to in the other groups treated with either treatment alone, but the differences were not significant. Bar represents standard error. **(C)** Kaplan-Meir survival curves of each groups. Survival rates were not significantly different. Abbreviations. Sim = simeprevir, RT = radiation.

In our recent study [6], the radiosensitizing effect of simeprevir was demonstrated in various human cancer cell lines. To investigate whether radiosensitization by simeprevir occurs *in vivo*, we constructed *in vivo* tumor xenograft models of BT474 breast cancer and U251 brain tumor cells, and the effects of simeprevir on tumor growth were investigated. Tumor volume curves for the control and groups treated with simeprevir, RT, or simeprevir plus RT are shown in Figure 1 and 2. In the breast cancer model (Figure 1), tumor growth was significantly delayed in the simeprevir plus RT group compared with the other three groups (p < 0.05). Differences in tumor growth were evident beginning at 13 days after grouping and persisted until the final measurement. Because all mice were alive at the end of the experiment, survival differences could not be assessed.

The effect of simeprevir on the brain tumor model is presented in Figure 2. Bioluminescence imaging (BLI) showed the greatest decrease in signals (Figure 2A) and the relative tumor burden was lowest in the simeprevir plus RT group (Figure 2B), although the differences were not significant. Additionally, the survival rates did not differ between groups (Figure 2C). According to our previous results, the radiosensitizing effect of simeprevir was evident *in vitro* in the U251 brain tumor cell line, but this was not observed in the *in vivo* model.

### Effect of PI4K IIIα inhibition on epithelial-mesenchymal transition

Epithelial-mesenchymal transition (EMT), an essential process for cancer development and progression, is known to be involved in activation of the PI3K/Akt pathway [22, 23]. We investigated the effect of PI4K IIIα inhibition by simeprevir on the EMT in a wound healing assay, modified Boyden chamber assay and based on vasculogenic mimicry (VM) formation.

**Figure 3:**
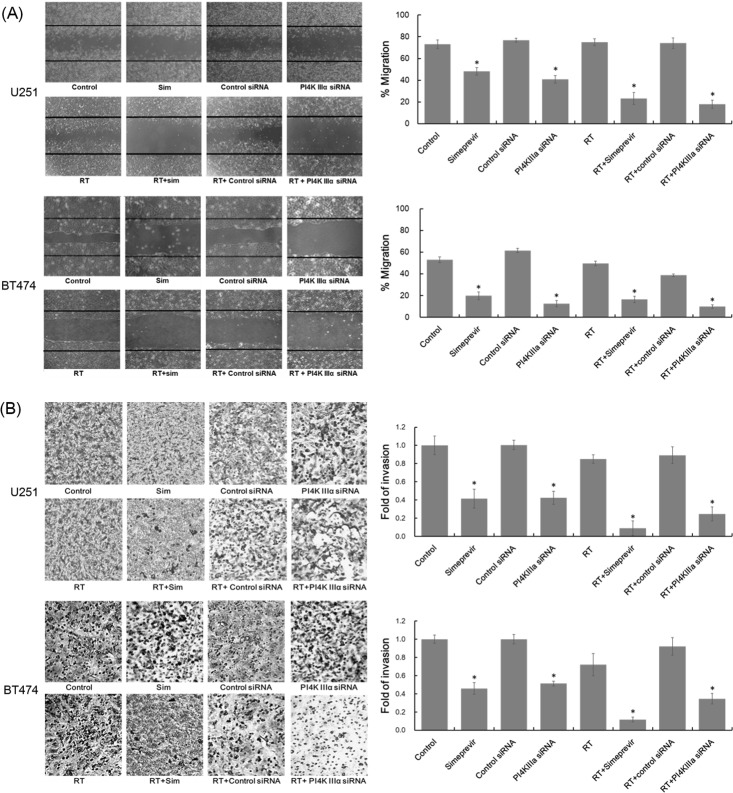
Effect of PI4K IIIα inhibition on invasion and migration Migration and invasion potential of U251 cells and BT474 cells were assessed with wound healing assays **(A)** and modified Boyden chamber assay **(B)**, respectively. Stained cells were analyzed in representative fields (x100). Selective inhibition of PI4K IIIα using RNAi inhibited cell migration and invasion in both cell lines and similar results were found after treatment with 200 nM simeprevir. The inhibition of cell migration and invasion was more prominent after treatment with simeprevir and radiation. Bar and asterisk represent standard error and statistical significance of p<0.05 compared with control, respectively. Abbreviations. Sim = simeprevir

Wound healing assays were performed in U251 and BT474 cell lines to evaluate the effects of PI4K IIIα inhibition on cell migration. Pretreatment with PI4K IIIα siRNA or simeprevir markedly inhibited the migration potential of both cancer cells (Figure 3A). The inhibitory effects of PI4K IIIα inhibition on migration potential were enhanced when combined with radiation. Similarly, the invasion rate of both cancer cell lines were significantly compromised after treatment with PI4K IIIα siRNA or simeprevir and the effects were more prominent when combined with radiation (Figure 3B).

VM is a special way of generating tumor vasculature without the involvement of endothelial cells and is an important feature of malignant cells linked to tumor growth and metastasis [24, 25]. Recently, the associations of VM with poor prognosis and metastasis in various cancers have been reported [26]. In this study, the inhibitory effect of simeprevir on VM was evaluated in a VM formation assay using U251 cells; the results showed that simeprevir significantly impaired VM formation compared to in the controls (Supplementary Figure 1).

### Radiosensitizing effect of PI4K IIIα inhibition was mediated by PI3Kδ inhibition

**Figure 4:**
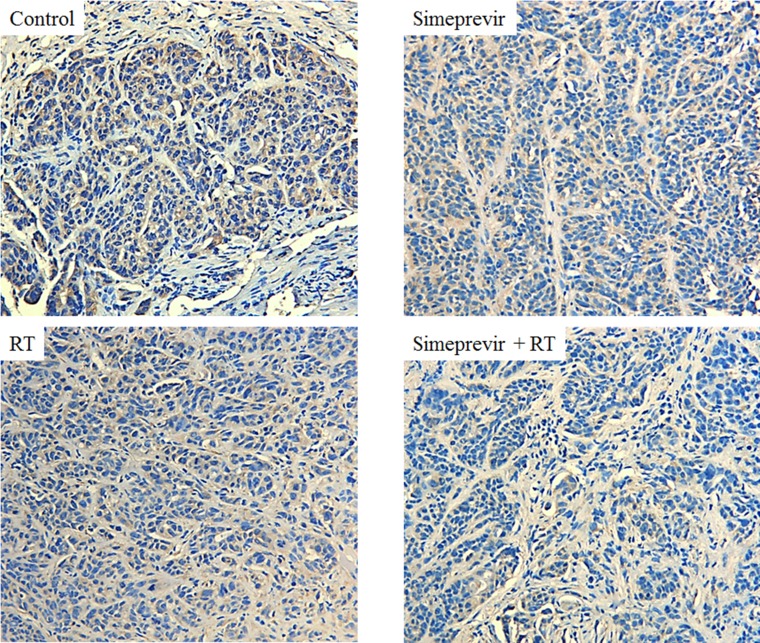
Immunohistochemical staining of *in vivo* breast tumor harvested from mice Expression of PI3Kδ was down-regulated in tumors treated with simeprevir plus radiation (x200). Abbreviations. RT = radiation.

Our previous study showed that the diverse mechanism of radiosensitization induced by PI4K IIIα inhibition down-regulated both the PI3K/Akt and PLC/PKC pathways [6]. Geng et al reported that a specific inhibitor of PI3Kδ enhanced radiation-induced cell killing both *in vitro* and *in vivo* [27]. Thus, we performed immunohistochemical staining using tissues harvested from a xenograft model to evaluate the effect of PI4K IIIα inhibition on the PI3Kδ isotype. We found that PI3Kδ expression was decreased in tumors treated with simeprevir plus RT compared to tumors from the other groups (Figure 4). This suggests that the radiosensitizing effect of PI4K IIIα inhibition is mediated by down-regulation of PI3Kδ.

**Figure 5:**
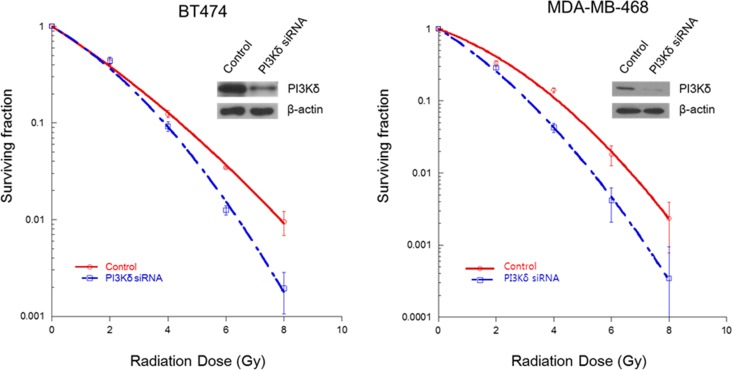
Radiosensitizing effect of PI3Kδ inhibition using RNAi Specific inhibition of PI3Kδ using RNA interference increased radiosensitivity of BT474 and MDA-MB-468 cells.

Next, to investigate the radiosensitizing effect of PI3Kδ inhibition, we performed a clonogenic assay using siRNA for PI3Kδ. As shown in Figure 5, specific inhibition of PI3Kδ using RNAi significantly enhanced radiation-induced cell killing in BT474 (SER_0.05_ 1.16) and MDA-MB-468 (SER_0.05_ 1.28) breast cancer cells.

### PI3Kδ inhibition via siRNA or simeprevir down-regulated PD-L1 expression

**Figure 6:**
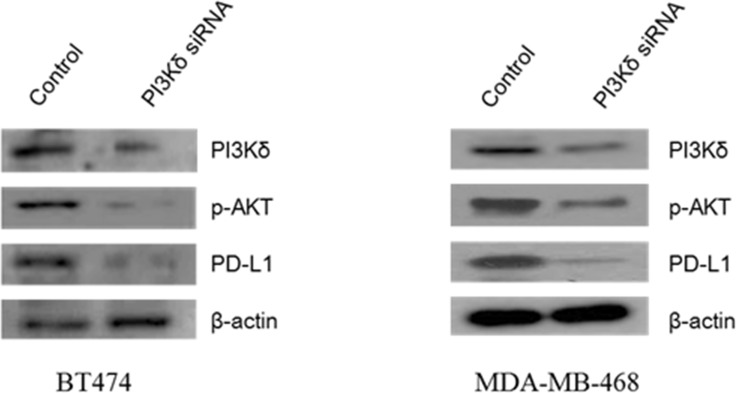
Effect of PI3Kδ inhibition on expression of phosphorylated (p)-AKT and PD-L1 in BT474 and MDA-MB-468 cells PI3Kδ inhibition using RNAi significantly decreased p-AKT expression in both cell lines and PD-L1 expression in MDA-MB-468 cells.

It has been reported that PI3Kδ has immune modulatory functions associated with PD-L1 expression [28, 29]. We evaluated whether PI3Kδ inhibition modulate PD-L1 expressions in BT474 and MDA-MB-468 breast cancer cells and assessed the mechanism of interaction. PI3Kδ inhibition using siRNA led to significant down-regulation of PD-L1 expression in MDA-MB-468 cells although the effect of PI3Kδ inhibition on PD-L1 expression was not prominent in BT474 cells. In both cell lines, PI3Kδ inhibition down-regulated the expression of p-Akt (Figure 6).

**Figure 7:**
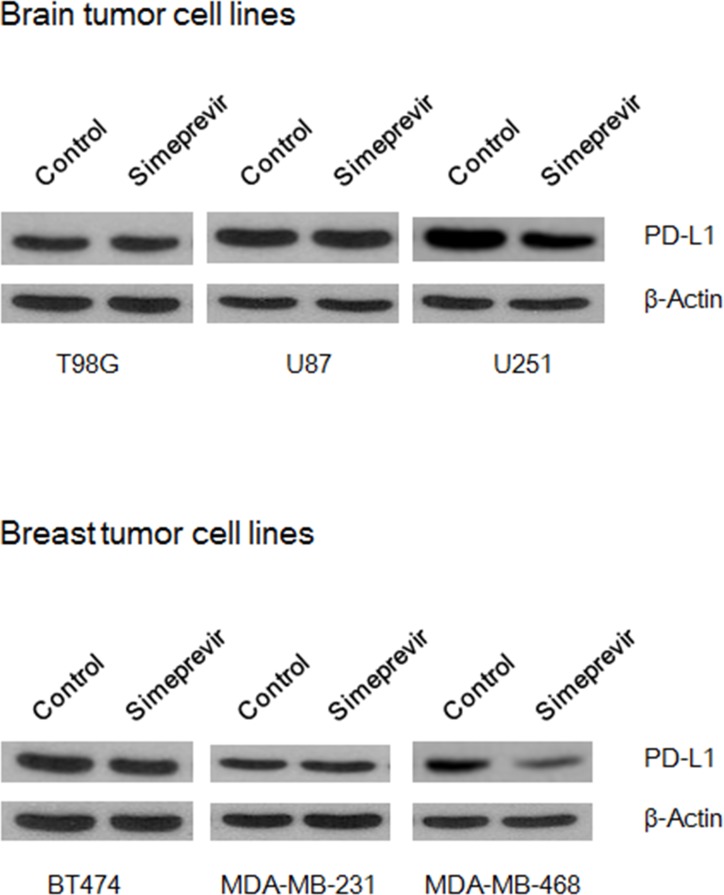
Effect of simeprevir on PD-L1 expressions in various brain and breast tumor cell lines In U251 and MDA-MB-468 cells, simeprevir (500 nM) significantly inhibited expression of PD-L1.

To determine whether PI3Kδ inhibition through PI4K IIIα inhibition affects PD-L1 expression, the effect of simeprevir on PD-L1 expression was evaluated in various cancer cell lines including T98G, U87, and U251 glioma cell lines and BT474, MDA-MB-231, and MDA-MB-468 breast cancer cell lines (Figure 7). In U251 and MDA-MB-468 cell lines, simeprevir significantly inhibited PD-L1 expression. It has been reported that the association of PD-L1 expression with poor prognosis was mainly found in triple negative breast cancer, [30, 31] and we speculated that simeprevir could effectively down-regulate the expression of PD-L1 in MDA-MB-468 cells exhibiting triple negative subtype. In glioma cells, the mechanism of PD-L1 expression has not been identified yet. We previously reported that the radiosensitizing effect of PI3K inhibitor on U251 was more prominent compared to other glioma cell lines [32]. As simeprevir affect the PD-L1 expression through modulation of the PI3Kδ, the effect of simeprevir would be more prominent in U251 cells than in the other cell lines. These results suggest that PI4K IIIα inhibition down-regulates PD-L1 expression by modulating PI3Kδ and p-Akt.

### Combination of PD-L1 inhibitor and PI4K IIIα inhibitior enhanced radiation-induced cell death through modulation of PI3Kδ

**Figure 8:**
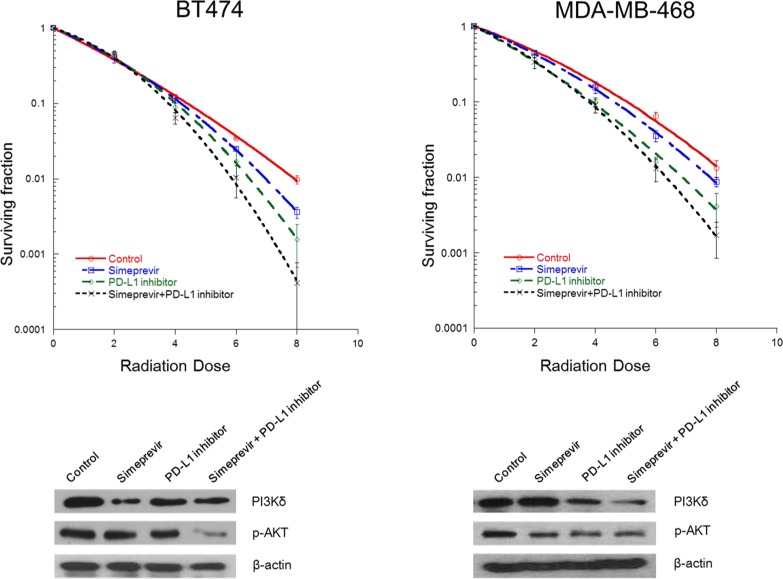
Effect of Simeprevir on radiosensitizing effect of PD-L1 blockade in BT474 and MDA-MB-468 cells Simeprevir and PD-L1 inhibitor alone increased radiation-induced cell death and down-regulated the expression of phosphorylated (p)-Akt. Combined treatment with the two agents showed additive radiosensitizing effects and down-regulation of p-Akt expression.

After the effects of PI4K IIIα on PD-L1 expressions were determined, we performed a clonogenic assay to assess the effect of PI4K IIIα on the radiosensitizing effect of immune checkpoint blockades in BT474 and MDA-MB-468 cells (Figure 8). Simeprevir combined with PD-L1 blockade significantly enhanced radiation-induced cell killing (SER_0.05_ 1.22 in BT 474 cells, 1.34 in MDA-MB-468 cells) compared to either treatment alone (SER_0.05_ for PI4KIIIα inhibitor and PD-L1 inhibitor, 1.13 and 1.08 in BT474 cells, 1.11 and 1.26 in MDA-MB-468 cells, respectively). PD-L1 inhibitor or PI4K IIIα inhibitor alone inhibited p-Akt expression in both cell lines as is shown in the results for PI3Kδ siRNA experiment; a combination of the two inhibitors led to the strongest down-regulation of p-Akt.

### Radiosensitizing and immune modulatory effect of PI4K IIIα inhibition in immune-competent syngeneic mouse model

**Figure 9:**
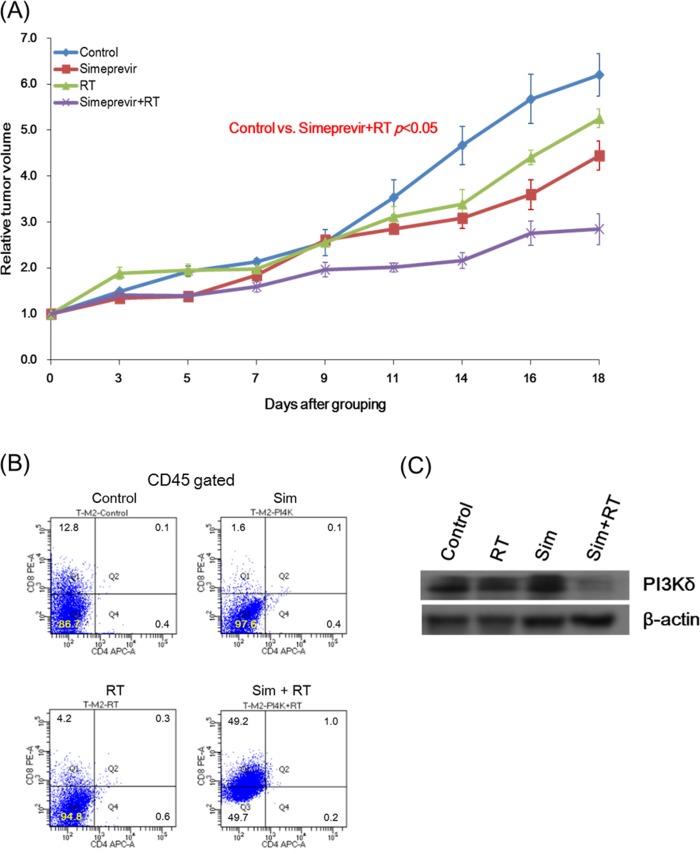
Radiosensitizing effect and immune modulatory function of simeprevir in immune-competent syngeneic mouse models Tumor volume curves showed significantly delayed tumor growth in the group treated with simeprevir and radiation in immune-competent syngeneic mouse model (P < 0.05). Relative tumor volume = tumor volume divided at specific time by tumor volume at randomization. Tumor cells harvested from mice were analyzed in fluorescence-activated cell sorting **(B)** and western blotting **(C)**. After treatment with simeprevir and radiation, the proportion of CD8(+) T cell was significantly increased and PI3Kδ expression was down-regulated. Abbreviations. Sim = simeprevir, RT = radiation.

To evaluate the immune modulatory effect of simeprevir, syngeneic 4T1 murine tumor model using immune-competent mice were constructed. The treatment groups were same as in the experiment of xenograft model; control, simeprevir, RT, or simeprevir plus RT. The simeprevir showed significant radiosensitizing effect in syngeneic murine tumor models (Figure 9A). To investigate the immune modulatory effect, flow cytometric analyses were performed using the tumor harvested from syngeneic mouse model. As a result (Figure 9B), the proportion of CD8(+) T cell was significantly increased after treatment with simeprevir combined with RT. PI3Kδ expression was down-regulated after treatment with simeprevir combined with RT (Figure 9C), suggesting that PI4K IIIα inhibition exhibit immune-modulation via down-regulation of PI3Kδ expression.

## DISCUSSION

Our previous *in vitro* study [6] reported the radiosensitizing effect of PI4K IIIα inhibition. Simeprevir, an anti-HCV agent, inhibited PI4K IIIα activity. Simeprevir increased radiation-induced cell killing in a panel of human cancer cell lines. Pretreatment with simeprevir led to prolongation of radiation-induced γH2AX foci and down-regulation of phospho-DNA-PKcs, indicating impaired DNA double-strand break repair. Apoptosis and autophagy were the major modes of cell death. Based on these results, we confirmed the radiosensitizing effect of PI4K IIIα inhibition in an *in vivo* tumor xenograft model in this study.

Immunotherapy is an emerging anti-cancer treatment, and enhanced cancer cell killing by radiotherapy when combined with immune checkpoint blockades have been reported [10, 33]. Although the specific mechanism of immune checkpoint blockades are not fully understood, some studies suggested that the PI3K pathway is involved in PD-L1 expression and immunoresistance in tumor cells [12, 34, 35]. We previously demonstrated that PI4K IIIα inhibition effectively down-regulated the PI3K pathway *in vitro*. Thus, we hypothesized the immune modulatory potential of PI4K IIIα. Therefore, we further investigated the mechanism of radiosensitization of PI4K IIIα inhibition, focusing on immune modulation and the tumor microenvironment.

In this study, we confirmed the radiosensitizing effect of PI4K IIIα inhibition using simeprevir in an *in vivo* tumor xenograft model. Combined treatment with simeprevir and radiation significantly attenuated tumor growth in a breast cancer xenograft model, while tumor growth in the radiation or simeprevir alone groups was similar to in the control group. This suggests that the dose of radiation delivered in our study, radiation therapy alone (9 Gy in 3 fractions), was not sufficient to delay tumor growth; however, when combined with simeprevir, the therapeutic efficacy of radiation was dramatically enhanced. A high dose of radiation increases tumor control, but toxicity to normal tissue also increases. Radiosensitizers were previously suggested to overcome this limitation, but the toxicity of radiosensitizers limited their widespread use. Simeprevir is an anti-HCV agent that has been shown to be safe in humans and is widely used to treat HCV without significant toxicity. Therefore, PI4K IIIα inhibition using simeprevir may be a viable and safe strategy for enhancing the cytotoxic effect of radiation therapy.

In a brain tumor xenograft model, the tendency for tumor growth delay with simeprevir was observed, but the results were not significant. In an *in vitro* experiment, simeprevir showed a significant radiosensitizing effect in U251 brain tumor cells [6]. Simeprevir was initially developed as an agent targeting HCV; the molecular weight is 749.9 Da (g/mol) and the blood-brain barrier (BBB) penetrability of simeprevir was not investigated. As is well-known, the BBB impairs effective delivery of a wide range of anti-cancer agents to the brain up to the therapeutic level. The less discernible radiosensitizing effect in the *in vivo* brain tumor model in this study may be explained by the limited penetration of simeprevir into the tumor in the brain because of its molecular weight. Although we delivered radiation before administration of simeprevir in the brain tumor model to increase the permeability of the BBB, a significant radiosensitizing effect was not observed. Further studies using an anti-PI4K IIIα agent that can cross the BBB up to a therapeutic level would confirm the *in vitro* radiosensitizing effect of PI4K IIIα inhibition in a brain tumor xenograft model.

The radiosensitizing effects of PI3K inhibition have been widely reported [32, 36–39]. Recently, a specific inhibitor of PI3Kδ, IC486068, was shown to enhance the radiosensitivity of tumor cells [27]. PI3Kδ, one of the isotypes in the class I PI3K family, which is expressed mainly in lymphoid cells, has been reported to be highly expressed in solid tumors as well as hematologic malignancies [20, 21]. Additionally, it was reported that overexpression of PI3Kδ resulted in constitutive Akt activation and induced oncogenic transformation of normal cells [40]. In our study, PI4K IIIα inhibition combined with radiation using simeprevir down-regulated PI3Kδ expression. Furthermore, specific inhibition of PI3Kδ using RNAi enhanced radiation-induced cell killing, suggested that the mechanism of radiosensitization is related in part to PI3Kδ inhibition. To our knowledge, this is the second study reporting the radiosensitizing effect of PI3Kδ; the first study was reported by Geng et al [27]. However, in their study, the suggested mechanism of radiosensitization was enhanced radiation-induced apoptosis of endothelial cells and impaired tumor vascular formation, but not a direct cytotoxic effect to cancer cells. Therefore, this is the first study reporting enhanced radiation-induced tumor cell killing by PI3Kδ inhibition, and PI3Kδ may be a potential target for radiosensitization by simultaneously regulating the radiation effect on tumor cells and tumor microenvironment.

The tumor microenvironment is known to be important in tumorigenesis and responsible for treatment resistance [41, 42]. EMT is an essential process for cancer cells to acquire invasiveness and metastatic potential and has been suggested as a promising target for modulating the tumor microenvironment [43, 44]. In our study, we found that PI4KIIIα inhibition led to eversion of EMT as indicated by impaired cell migration/invasion and VM formation. Geng et al. showed that that PI3Kδ inhibited the tumor vasculature by enhancing radiation-induced cell death of endothelial cells and inhibited endothelial cell migration [27]. Another study by Sawyer et al. demonstrated that PI3Kδ is associated with breast cancer cell migration, regulating both directionality and migration speed, and confers selective migratory capacities (chemotaxis) of breast cancer cells [20]. Therefore, we predict that PI4K IIIα inhibition via PI3Kδ inhibition controls the eversion of EMT.

Previously, Ali et al. showed that PI3Kδ inactivation inhibited tumor growth in an immune-competent syngeneic murine tumor model through enhanced cytotoxic T cell functions by disrupting regulatory T cell function, which is known to be essential for immune evasion of tumors [28]. It also has been demonstrated that PI3Kδ is required for cytotoxic T cell functions involving the proliferation of cytotoxic T cells and degranulation process [45]. Kan-o et al. [29] reported that PI3Kδ upregulated PD-L1 in airway epithelial cells, which is an immune evasion strategy used by respiratory viruses. Based on these reports, the role of PI3Kδ in immune modulation is suggested, but direct evidence showing an association between PI3Kδ and immune-modulating markers in cancer have not been reported. In our study, PD-L1 expression was decreased in the presence of the PI4K IIIα inhibitor and following specific inhibition of PI3Kδ using RNAi. This is the first study to demonstrate the association of PI3Kδ with PD-L1 in tumor cells. Furthermore, PI4K IIIα inhibitor enhanced the radiosensitizing effect of PD-L1 blockade. Therefore, the immune modulatory function of PI4K IIIα inhibitor and complimentary role of PD-L1 function is suggested and the modulation of PI3Kδ appears to be the underlying mechanism.

Although we investigated immune-modulating function via *in vitro* human cancer cells and human T lymphocytes co-culture system, the findings provided only limited mechanistic interpretation. To overcome this issue, we performed *in vivo* study using immune-competent syngeneic murine tumor models. PI4K IIIα inhibition using simeprevir showed significant radiosensitizing effect in this syngeneic murine tumor model. When evaluating the tumor cells harvested from murine tumor sample, the proportion of CD8(+) T lymphocyte was significantly increased while that of CD4(+) T lymphocyte was not significantly changed. Many studies have been reported the association of tumor infiltrating CD8(+) lymphocytes with prognosis [46, 47] and prognostic value of CD4(+)/CD8(+) ratio of tumor infiltrating lymphocytes [48, 49]. We can speculate that simeprevir can increase anticancer immune response by affecting the CD4(+)/CD8(+) ratio of T lymphocyte.

In conclusion, we confirmed the radiosensitizing effect of PI4K IIIα inhibition using simeprevir in *in vivo* tumor xenograft models and immune-competent syngeneic mouse models and investigated the mechanism of the interaction focusing on immune modulation and EMT. The PI4K IIIα inhibitor radiosensitized tumor cells by modulating PI3Kδ and regulated the immune response by regulating PD-L1 expression and affecting the CD4(+)/CD8(+) ratio of T lymphocyte. PI4K IIIα inhibition led to eversion of EMT features. Taken together, targeting of PI4K IIIα may enhance the cytotoxic effect of radiation by modulating anti-tumor immunity and the tumor microenvironment.

## MATERIALS AND METHODS

### Cell culture

The human breast cancer cell line (BT474, MDA-MB-231 and MDA-MB-468), human malignant glioma cell line (T98G, U87, U251) and murine breast cancer cell line (4T1) purchased from the American Type Culture Collection (Manassas, VA, USA) were cultured at 37°C in 5% CO_2_ humidified chambers. Cells were maintained in Dulbecco Modified Eagle Medium (Life Technology, Inc., Carlsbad, CA, USA) supplemented with 10% fetal bovine serum or Roswell Park Memorial Institute 1640 (Life Technology, Inc.) at 37°C in 5% CO_2_ using standard techniques. When evaluating the immune modulatory effect *in vitro*, the tumor cells were co-cultured with activated Jurkat T cells.

### *In vivo* tumor xenograft model

#### Breast tumor model

By injecting 5 × 10^6^ BT474 cells into the hind limb of 6- to 8-week-old BALB/c athymic nude mice (Orient Bio, Inc., Sungnam, Korea), human breast cancer xenografts were established. All procedures were performed according to a protocol approved by the Institutional Animal Care and Use Committee at the Clinical Research Institute, Seoul National University Bundang Hospital. Tumor growth was monitored weekly and tumor volume was estimated using the formula (length × width × width)/2. On day 25 after injection, mice were randomized into 4 groups, control, simeprevir, radiation (RT), and simeprevir plus RT. Simeprevir (10 mg/kg) was administered intraperitoneally three times weekly for 2 weeks. During the second week of drug treatment, mice were irradiated three times with 3 Gy for a total of 9 Gy using a 6 MeV electron beam with a 1 cm bolus applied 3 h after simeprevir injection. Lead blocks were used to shield the non-tumor part of the mice. After treatment was complete, tumor size was measured weekly. Mice were sacrificed at 3 months after tumor inoculation and tumors were harvested.

#### Brain tumor model

U251 cells were transfected with a pGL4.5 luciferase reporter vector (Promega, Madison, WI, USA). U251 cells (3 × 10^5^ cells) were injected into the caudate-putamen of BALB/c athymic nude mice with a 26-G needle attached to a Hamilton syringe and the human brain tumor xenograft model was established. Two weeks after injection, mice were randomized into 4 groups: control, simeprevir, RT, and simeprevir plus RT. The dose and administration schedule of simeprevir and radiation were same as in the breast tumor xenograft model. To enable simeprevir to cross the BBB, RT was administered before simeprevir injection.

#### Syngeneic mouse model

The 4T1 murine triple negative breast cancer cells (6 × 10^5^ cells) were injected into the hind limb of 6- to 8-week-old BALB/c immune-competent mice (Orient Bio, Inc). After 12 days after injection, mice were randomized into 4 groups; control, simeprevir, radiation (RT), and simeprevir plus RT. Simeprevir (20mg/kg) and radiation (8 Gy × 3 fractions) were delivered in the same way as previously described in *in vivo* xenograft model. Mice were sacrificed at 1 month after tumor inoculation and tumors were harvested.

### Bioluminescence imaging (BLI)

Bioluminescence imaging was performed using the IVIS Imaging System 100 series (Xenogen Corporation, Alameda, CA, USA) according to the manufacturer's protocol. Mice were injected with 150 mg/kg D-luciferin intraperitoneally and after 15 min, anesthetized with 1–2% isoflurane. Images were acquired between 5 and 10 min and peak luminescence signals were recorded. To compensate initial small differences in tumor burdens, the relative tumor burdens were defined as (signal value at the time of last measurement – signal value at baseline)/signal value at baseline; these values were used for statistical analysis.

### Immunohistochemical (IHC) staining

The tissues harvested from mice were fixed with formalin and paraffin-embedded tissue blocks were constructed. Tissue sections were deparaffinized and rehydrated according to standard procedures. Staining was conducted using antibodies against PI3Kdelta (Abcam, Cambridge, UK).

### Wound healing assay

Cells were grown to confluence in 6-well plates (Sonic-Seal Slide, Nalgene Nunc, Rochester, NY, USA) and then starved by culturing in DMEM without fetal bovine serum for 24 hour. Each well was then divided into a 2 × 3 grid. Using a 1-mL pipette tip, a line was scratched in each hemisphere of the well to wound the cells, and the medium was changed to starvation medium. Images were taken of the intersections of linear cell wounds and grid lines. Images were captured immediately after wounding and after 24 h. The distance between wound edges was measured using Image J software (NIH, Bethesda, MD, USA).

### Modified boyden chamber assay

A Transwell system (Corning, Rochester, NY, USA), which allows cells to migrate through 8 μm pores in polycarbonate membranes was used to evaluate the cell invasion. After trypsinization, an aliquot of 104 cells was added to the upper chamber. After 24 hours, inserts were fixed in methanol and stained with 1% crystal violet. The invading cells on the lower surface were counted to calculate the invasion.

### Vasculogenic mimicry (VM) assay

A commercial Matrigel assay kit (BD Biosciences, Franklin Lakes, NJ, USA) was used to assess VM. Extracellular matrix matrigel (200 μL) was placed in the wells of 48-well plates and incubated at 37°C for 2 h. Cells were treated with simeprevir and then seeded onto the coated plate. After incubation for 24 h, VM was assessed using an inverted microscope.

### RNA interference

Cells were plated in 6-well culture plates and transfected with PI3Kδ-specific (GE Dharmacon, Lafayette, CO, USA) or nonspecific RNA using Lipofectamine RNAiMAX transfection reagent (Invitrogen, Carlsbad, CA, USA) in reduced-serum medium (OPTIMEM, Life Technologies) was used according to the manufacturer's protocol.

### Clonogenic assay

Identical numbers of cells were plated into 6-well culture plate across the different treatment groups for each radiation dose. Forty-eight hours after treatment, the cells were irradiated with 6 megavoltage X-ray from a linear accelerator (Varian Medical System, Palo Alto, CA, USA) at a dose rate of 2.46 Gy/min and then incubated for colony formation for 14–23 days. Colonies were fixed with methanol and stained with 0.5% crystal violet. The colonies containing 50 or more cells at least were counted and the surviving fraction was calculated. The radiation-survival data was fitted to a linear-quadratic model using Kaleidagraph version 3.51 (Synergy Software, Reading, PA, USA). A sensitizer enhancement ratio of 0.05 (SER_0.05_) was calculated as the ratio of the dose resulting in a surviving fraction of 0.05 without drugs to that with drugs.

### Western blot analysis

Cells were washed, scraped, and resuspended in lysis buffer (Cell Signaling Technology, Danvers, MA, USA). Proteins were solubilized by sonication, separated by SDS-PAGE, and electroblotted onto polyvinylidenedifluoride membranes (Millipore). Membranes were blocked in Tris-buffered saline and Tween-20 solution containing 5% skim milk or bovine serum albumin and then probed with a primary antibody directed against PI3Kδ (Abcam), p-AKT (Cell Signaling Technology), PD-L1 (Biolegend, San Diego, CA, USA), and β-actin (Santa Cruz Biotechnology, Santa Cruz, CA, USA). After washing and blocking, membranes were incubated with peroxidase-conjugated goat anti-rabbit or anti-mouse IgG secondary antibody (Jackson ImmunoResearch Laboratories, West Grove, PA, USA) at dilutions of 1:10,000 for 1 h.

### Flow cytometry analysis

Tumor cells were dissociated into single cell suspension and stained with CD45, CD4 and CD8 antibodies. Cells were analyzed and sorted using a FACS Calibur flow cytometer (BD Biosciences) and FACS Aria sorters (BD Biosciences).

### Statistical analysis

Statistical analysis was performed with SPSS version 18.0 (SPSS, Inc., Chicago, IL, USA). The *t*-test and one way analysis of variance were used to compare the mean values between groups. Survival differences were calculated using the Kaplan-Meier method and statistical significance were assessed with the log-rank test.

## SUPPLEMENTARY MATERIALS FIGURE



## References

[1] Clayton EL, Minogue S, Waugh MG (2013). Mammalian phosphatidylinositol 4-kinases as modulators of membrane trafficking and lipid signaling networks. Prog Lipid Res.

[2] Waugh MG (2012). Phosphatidylinositol 4-kinases, phosphatidylinositol 4-phosphate and cancer. Cancer Lett.

[3] Ishikawa S, Egami H, Kurizaki T, Akagi J, Tamori Y, Yoshida N, Tan X, Hayashi N, Ogawa M (2003). Identification of genes related to invasion and metastasis in pancreatic cancer by cDNA representational difference analysis. J Exp Clin Cancer Res.

[4] Ilboudo A, Nault JC, Dubois-Pot-Schneider H, Corlu A, Zucman-Rossi J, Samson M, Le Seyec J (2014). Overexpression of phosphatidylinositol 4-kinase type IIIα is associated with undifferentiated status and poor prognosis of human hepatocellular carcinoma. BMC Cancer.

[5] Sbrissa D, Semaan L, Yanfeng L, Shisheva A, Chinni SR (2015). Abstract 5162: phosphatidylinositol 4-kinase type IIIa (PI4KA) expression in prostate cancer. Cancer Res.

[6] Kwon J, Kim DH, Park JM, Park YH, Hwang YH, Wu HG, Shin KH, Kim IA (2016). Targeting phosphatidylinositol 4-kinase IIIα for radiosensitization: a potential model of drug repositioning using anti-HCV agent. Int J Radiat Oncol Biol Phys.

[7] Mahoney KM, Rennert PD, Freeman GJ (2015). Combination cancer immunotherapy and new immunomodulatory targets. Nat Rev Drug Discov.

[8] Chen J, Jiang CC, Jin L, Zhang XD (2016). Regulation of PD-L1: a novel role of pro-survival signalling in cancer. Ann Oncol.

[9] Sharabi AB, Lim M, DeWeese TL, Drake CG (2015). Radiation and checkpoint blockade immunotherapy: radiosensitisation and potential mechanisms of synergy. Lancet Oncol.

[10] Deng L, Liang H, Burnette B, Beckett M, Darga T, Weichselbaum RR, Fu YX (2014). Irradiation and anti–PD-L1 treatment synergistically promote antitumor immunity in mice. J Clin Invest.

[11] Parsa AT, Waldron JS, Panner A, Crane CA, Parney IF, Barry JJ, Cachola KE, Murray JC, Tihan T, Jensen MC, Mischel PS, Stokoe D, Pieper RO (2007). Loss of tumor suppressor PTEN function increases B7-H1 expression and immunoresistance in glioma. Nat Med.

[12] Atefi M, Avramis E, Lassen A, Wong DJ, Robert L, Foulad D, Cerniglia M, Titz B, Chodon T, Graeber TG (2014). Effects of MAPK and PI3K pathways on PD-L1 expression in melanoma. Clin Cancer Res.

[13] Song M, Chen D, Lu B, Wang C, Zhang J, Huang L, Wang X, Timmons CL, Hu J, Liu B, Wu X, Wang L, Wang J, Liu H (2013). Pten loss increases PD-L1 protein expression and affects the correlation between PD-L1 expression and clinical parameters in colorectal cancer. PLoS One.

[14] Fung-Leung WP (2011). Phosphoinositide 3-kinase delta (PI3Kδ) in leukocyte signaling and function. Cellular Signal.

[15] Chantry D, Vojtek A, Kashishian A, Holtzman DA, Wood C, Gray PW, Cooper JA, Hoekstra MF (1997). P110delta, a novel phosphatidylinositol 3-kinase catalytic subunit that associates with p85 and is expressed predominantly in leukocytes. J Biol Chem.

[16] Burger JA, Okkenhaug K (2014). Haematological cancer: idelalisib[mdash]targeting PI3K[delta] in patients with B-cell malignancies. Nat Rev Clin Oncol.

[17] Gopal AK, Kahl BS, de Vos S, Wagner-Johnston ND, Schuster SJ, Jurczak W, Flinn IW, Flowers CR, Martin P, Viardot A (2014). Mature follow up from a phase 2 study of PI3K-delta inhibitor idelalisib in patients with double (rituximab and alkylating agent)-refractory indolent B-cell non-hodgkin lymphoma (iNHL). Blood.

[18] O'Connor OA, Flinn IW, Patel MR, Fenske TS, Deng C, Brander DM, Gutierrez M, Jones S, Kuhn JG, Miskin HP (2015). TGR-1202, a novel once daily PI3K-delta inhibitor, demonstrates clinical activity with a favorable safety profile in patients with CLL and B-cell lymphoma. Blood.

[19] Yang Q, Modi P, Newcomb T, Quéva C, Gandhi V (2015). Idelalisib: first-in-class PI3K delta inhibitor for the treatment of chronic lymphocytic leukemia, small lymphocytic leukemia, and follicular lymphoma. Clin Cancer Res.

[20] Sawyer C, Sturge J, Bennett DC, O’Hare MJ, Allen WE, Bain J, Jones GE, Vanhaesebroeck B (2003). Regulation of breast cancer cell chemotaxis by the phosphoinositide 3-kinase p110δ. Cancer Res.

[21] Tzenaki N, Andreou M, Stratigi K, Vergetaki A, Makrigiannakis A, Vanhaesebroeck B, Papakonstanti EA (2012). High levels of p110δ PI3K expression in solid tumor cells suppress PTEN activity, generating cellular sensitivity to p110δ inhibitors through PTEN activation. FASEB J.

[22] Larue L, Bellacosa A (2005). Epithelial-mesenchymal transition in development and cancer: role of phosphatidylinositol 3′ kinase/AKT pathways. Oncogene.

[23] Xu W, Yang Z, Lu N (2015). A new role for the PI3K/Akt signaling pathway in the epithelial-mesenchymal transition. Cell Adh Migr.

[24] Folberg R, Hendrix MJ, Maniotis AJ (2000). Vasculogenic mimicry and tumor angiogenesis. Am J Pathol.

[25] Dunleavey JM, Dudley AC (2012). Vascular mimicry: concepts and implications for anti-angiogenic therapy. Curr Angiogenes.

[26] Cao Z, Bao M, Miele L, Sarkar FH, Wang Z, Zhou Q (2013). Tumour vasculogenic mimicry is associated with poor prognosis of human cancer patients: a systemic review and meta-analysis. Eur J Cancer.

[27] Geng L, Tan J, Himmelfarb E, Schueneman A, Niermann K, Fu A, Cuneo K, Kesicki EA, Treiberg J, Hayflick JS (2004). A specific antagonist of the p110δ catalytic component of phosphatidylinositol 3′-kinase, IC486068, enhances radiation-induced tumor vascular destruction. Cancer Res.

[28] Ali K, Soond DR, Piñeiro R, Hagemann T, Pearce W, Lim EL, Bouabe H, Scudamore CL, Hancox T, Maecker H (2014). Inactivation of PI (3) K p110 [dgr] breaks regulatory T-cell-mediated immune tolerance to cancer. Nature.

[29] Kan-o K, Matsumoto K, Asai-Tajiri Y, Fukuyama S, Hamano S, Seki N, Nakanishi Y, Inoue H (2013). PI3K-delta mediates double-stranded RNA-induced upregulation of B7-H1 in BEAS-2B airway epithelial cells. Biochem Biophys Res Commun.

[30] Kim HM, Lee J, Koo JS (2017). Clinicopathological and prognostic significance of programmed death ligand-1 expression in breast cancer: a meta-analysis. BMC Cancer.

[31] Mittendorf EA, Philips AV, Meric-Bernstam F, Qiao N, Wu Y, Harrington S, Su X, Wang Y, Gonzalez-Angulo AM, Akcakanat A, Chawla A, Curran M, Hwu P (2014). PD-L1 expression in triple-negative breast cancer. Cancer Immunol Res.

[32] Choi EJ, Cho BJ, Lee DJ, Hwang YH, Chun SH, Kim HH, Kim IA (2014). Enhanced cytotoxic effect of radiation and temozolomide in malignant glioma cells: targeting PI3K-akt-mtor signaling, HSP90 and histone deacetylases. BMC Cancer.

[33] Dovedi SJ, Adlard AL, Lipowska-Bhalla G, McKenna C, Jones S, Cheadle EJ, Stratford IJ, Poon E, Morrow M, Stewart R (2014). Acquired resistance to fractionated radiotherapy can be overcome by concurrent PD-L1 blockade. Cancer Res.

[34] Crane C, Panner A, Murray J, Wilson S, Xu H, Chen L, Simko J, Waldman F, Pieper R, Parsa A (2009). PI (3) kinase is associated with a mechanism of immunoresistance in breast and prostate cancer. Oncogene.

[35] Jiang X, Zhou J, Giobbie-Hurder A, Wargo J, Hodi FS (2013). The activation of mapk in melanoma cells resistant to braf inhibition promotes PD-L1 expression that is reversible by mek and PI3K inhibition. Clin Cancer Res.

[36] No M, Choi EJ, Kim IA (2009). Targeting HER2 signaling pathway for radiosensitization: alternative strategy for therapeutic resistance. Cancer Biol Ther.

[37] Jang NY, Kim DH, Cho BJ, Choi EJ, Lee JS, Wu HG, Chie EK, Kim IA (2015). Radiosensitization with combined use of olaparib and PI-103 in triple-negative breast cancer. BMC Cancer.

[38] Toulany M, Rodemann HP (2015). Phosphatidylinositol 3-kinase/Akt signaling as a key mediator of tumor cell responsiveness to radiation. Semin Cancer Biol.

[39] Toulany M, Iida M, Keinath S, Iyi FF, Mueck K, Fehrenbacher B, Mansour WY, Schaller M, Wheeler DL, Rodemann HP (2016). Dual targeting of PI3K and MEK enhances the radiation response of K-RAS mutated non-small cell lung cancer. Oncotarget.

[40] Kang S, Denley A, Vanhaesebroeck B, Vogt PK (2006). Oncogenic transformation induced by the p110β,-γ, and-δ isoforms of class I phosphoinositide 3-kinase. Proceedings of the National Academy of Sciences.

[41] Heinrich EL, Walser TC, Krysan K, Liclican EL, Grant JL, Rodriguez NL, Dubinett SM (2012). The inflammatory tumor microenvironment, epithelial mesenchymal transition and lung carcinogenesis. Cancer Microenviron.

[42] Gao D, Vahdat LT, Wong S, Chang JC, Mittal V (2012). Microenvironmental regulation of epithelial-mesenchymal transitions in cancer. Cancer Res.

[43] Creighton CJ, Gibbons DL, Kurie JM (2013). The role of epithelial-mesenchymal transition programming in invasion and metastasis: a clinical perspective. Cancer Manag Res.

[44] Jung HY, Fattet L, Yang J (2015). Molecular pathways: linking tumor microenvironment to epithelial-mesenchymal transition in metastasis. Clin Cancer Res.

[45] Putz EM, Prchal-Murphy M, Simma OA, Forster F, Koenig X, Stockinger H, Piekorz RP, Freissmuth M, Muller M, Sexl V, Zebedin-Brandl E (2012). PI3Kdelta is essential for tumor clearance mediated by cytotoxic T lymphocytes. PLoS One.

[46] Hadrup S, Donia M, Thor Straten P (2013). Effector CD4 and CD8 T cells and their role in the tumor microenvironment. Cancer Microenviron.

[47] Mahmoud SM, Paish EC, Powe DG, Macmillan RD, Grainge MJ, Lee AH, Ellis IO, Green AR (2011). Tumor-infiltrating CD8+ lymphocytes predict clinical outcome in breast cancer. J Clin Oncol.

[48] Diederichsen AC, Hjelmborg Jv, Christensen PB, Zeuthen J, Fenger C (2003). Prognostic value of the CD4+/CD8+ ratio of tumour infiltrating lymphocytes in colorectal cancer and HLA-DR expression on tumour cells. Cancer Immunol Immunother.

[49] Shah W, Yan X, Jing L, Zhou Y, Chen H, Wang Y (2011). A reversed CD4/CD8 ratio of tumor-infiltrating lymphocytes and a high percentage of CD4(+)FOXP3(+) regulatory T cells are significantly associated with clinical outcome in squamous cell carcinoma of the cervix. Cell Mol Immunol.

