# Supplementary material for: Inhibition of PI4K IIIα radiosensitizes in human tumor xenograft and immune-competent syngeneic murine tumor model
Source: Oncotarget. 2017 Nov 30;8(66):110392–405. doi: 10.18632/oncotarget.22778 (PMC5746391; doi:10.18632/oncotarget.22778)
Supplement: Supplementary file 1 [file oncotarget-08-110392-s001.pdf]

Inhibition of PI4K IIIα radiosensitizes in human tumor xenograft and immune-competent syngeneic murine tumor model

SUPPLEMENTARY MATERIALS

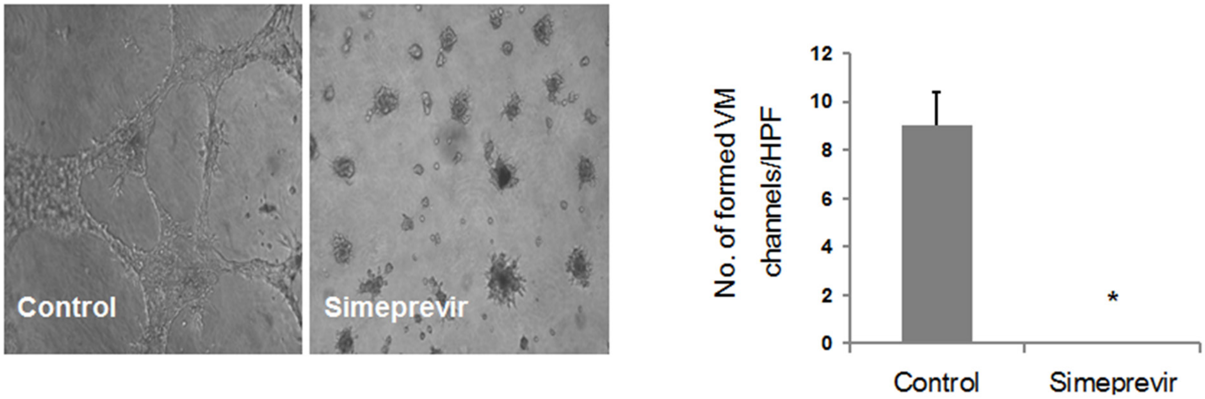

Supplementary Figure 1: Effect of simeprevir on vasculogenic mimicry (VM) formation. VM formation assay showed significant inhibition of VM formation in U251 cells. The ability of cells to form VM when plated on Matrigel was determined. After treatment with simeprevir, an inhibitory effect was observed based on photographs of representative VM formation fields (x 200).
